# Predictive Risk Factors Associated with Severe Radiation-Induced Mucositis in Nasopharyngeal or Oropharyngeal Cancer Patients: A Retrospective Study

**DOI:** 10.3390/biomedicines10102661

**Published:** 2022-10-21

**Authors:** Yumiko Kawashita, Sakiko Soutome, Masahiro Umeda, Toshiyuki Saito

**Affiliations:** 1 Department of Oral Health, Nagasaki University Graduate School of Biomedical Sciences, Nagasaki 852-8102, Japan; 2 Department of Clinical Oral Oncology, Nagasaki University Graduate School of Biomedical Sciences, Nagasaki 852-8102, Japan

**Keywords:** head and neck cancer, radiation-induced mucositis, retrospective study, risk factor

## Abstract

Radiation-induced mucositis in head and neck cancer patients generates difficulties in eating and swallowing, and may influence treatment tolerance, compliance, and quality of life. However, predictive factors have not been studied in detail. Thus, the aim of this study was to describe the association between pre-radiotherapy clinical factors and the incidence of severe radiation-induced mucositis in nasopharyngeal or oropharyngeal cancer patients. This retrospective study included all patients with definitive radiotherapy or chemoradiotherapy for nasopharyngeal or oropharyngeal cancer between July 2011 and June 2021 in a single center. The eligibility criteria included patients who received oral management during radiotherapy. Exclusion criteria was patients who received postoperative radiotherapy. The data were acquired from the medical records of patients. One hundred patients were included in this retrospective study. Grade 3 radiation-induced mucositis occurred in 47 patients (47%). Lymphocyte count was significantly associated with grade 3 mucositis (OR = 0.40; 95% CI = 0.19–0.86; *p* = 0.018). It is suggested that pre-radiation lower lymphocyte counts are a predictive risk factor for severe mucositis in patients who undergo definitive radiotherapy or chemoradiotherapy for nasopharyngeal or oropharyngeal cancer

## 1. Introduction

Standard therapies for head and neck cancer are surgery, chemotherapy, and radiotherapy [1]. Radiotherapy, unlike surgery, has the advantage of organ preservation. However, radiotherapy induces severe adverse events in both the acute and late phases. Since acute adverse events are generally transient and technically difficult to prevent, events such as mucositis have had less focus in research. However, radiation-induced mucositis generates difficulties in eating and swallowing, and may influence treatment tolerance, compliance, and quality of life.

We can predict the extent of radiation-induced mucositis according to the radiation dose-distribution diagram. Intensity-modulated radiotherapy (IMRT) has been used instead of three-dimensional conformal radiotherapy (3D-CRT) to treat almost all head and neck cancer patients. When 3D-CRT is used, the risk of radiation-induced mucositis is directly associated with the prescribed dose and size of the radiotherapy field. However, the field size and dose information are not useful for the prediction of radiation-induced mucositis in IMRT treatment, since the radiation intensity within the field varies. Hence, the entire 3D dose should be evaluated [2].

Clinical risk factors for radiation-induced mucositis have been studied. Treatment with pilocarpine has been suggested to reduce the risk of severe mucositis in oral cancer patients [3]. Oral hygiene has been significantly associated with radiation-induced oral mucositis [4]. Thus, in our previous study, we selected hypopharyngeal and laryngeal cancer patients whose radiation-induced mucositis was not influenced by teeth [5] or xerostomia. As a result, pre-radiotherapy, the neutrophil-to-lymphocyte ratio (NLR) was significantly associated with severe radiation-induced mucositis [6].

The aim of this study was to describe the association between pre-radiotherapy clinical factors and the incidence of severe radiation-induced mucositis in nasopharyngeal or oropharyngeal cancer patients.

## 2. Materials and Methods

### 2.1. Setting and Design

This retrospective study included all patients who received definitive radiotherapy or chemoradiotherapy (CRT) for nasopharyngeal or oropharyngeal cancer at Nagasaki University Hospital, Japan. The eligibility criteria were patients who received oral management between July 2011 and June 2021. The exclusion criteria were patients who received postoperative radiotherapy, because the radiation dose for postoperative radiotherapy is lower than that for definitive radiotherapy or CRT. The data were acquired from the medical records of patients.

### 2.2. Ethical Statement

The trial was conducted in accordance with the Declaration of Helsinki as revised in 2013. The study was approved by the Institutional Review Board (IRB) of Nagasaki University Hospital (approval number: 22011710; 25 January 2022). The need for informed consent was waived by the Nagasaki University Hospital Clinical Research Ethics Committee because of the retrospective nature of the study. Moreover, the research plan was published on the homepage of the participating hospitals according to the instructions of the IRB in accordance with the guaranteed opt-out opportunity.

### 2.3. Outcome

The severity of radiation-induced nasopharyngeal or oropharyngeal mucositis was graded according to the National Cancer Institute Common Terminology Criteria for Adverse Events Version 5.0 (CTC v5.0). The nearest match to the grade specified in the CTC v.5.0 was used. The highest grade of the severity of the mucositis during radiotherapy was recorded.

The severity of oral mucositis was as follows: grade 1, asymptomatic or mild symptoms, intervention not indicated; grade 2, moderate pain or ulcer that does not interfere with oral intake, modified diet indicated; grade 3, severe pain, interfering with oral intake; grade 4, life-threatening consequences, urgent intervention indicated; and grade 5, death. The severity of pharyngeal mucositis was as follows: grade 1, endoscopic findings only, minimal symptoms with normal intake, mild pain but analgesics not indicated; grade 2, moderate pain, analgesics indicated, altered oral intake, limiting instrumental activities of daily living (ADL); grade 3, severe pain; unable to adequately aliment or hydrate orally, limiting self-care ADL; grade 4, life-threatening consequences, urgent operative intervention indicated; and grade 5, death.

### 2.4. Characteristics

The following demographic and clinical characteristics were collected from medical records: demographic factors (age, sex, body mass index (BMI), Eastern Cooperative Oncology Group performance status (ECOG PS), and diabetes mellitus); tumor-related factors (primary tumor location, histopathology, and stage); treatment-related factors (concomitant CRT, radiation method, and total radiation dose); laboratory test results (neutrophil and lymphocyte counts and concentrations of serum albumin, hemoglobin, and serum creatinine immediately before radiotherapy); and completion of radiotherapy. ECOG PS score was as follows: score 0, fully active, able to carry on all pre-disease performance without restriction; score 1, restricted in physically strenuous activity but ambulatory and able to carry out work of a light or sedentary nature, e.g., light housework, office work; score 2, ambulatory and capable of all self-care but unable to carry out any work activities, up and about more than 50% of waking hours; score 3, capable of only limited self-care, confined to bed or chair more than 50% of waking hours; and score 4, completely disabled, cannot carry on any self-care, totally confined to bed or chair. The NLR was calculated by dividing the neutrophil count by the lymphocyte count. Concomitant CRT was categorized as none (radiotherapy alone), cetuximab (bioradiotherapy (BRT)), and cisplatin or carboplatin CRT. The radiation method was categorized as 3D-CRT or IMRT. Completion of radiotherapy was categorized into three groups: completion; discontinuation; and treatment pause.

### 2.5. Oral Management Associated with Radiotherapy

All of the patients received oral management prior to the commencement of radiotherapy [5,7]. Oral and dental evaluations, including panoramic X-ray examinations, were performed immediately after doctor introduction, and infected teeth were extracted to prevent osteoradionecrosis. During radiotherapy, the teeth in dentulous patients were covered by spacers to prevent scatter radiation, particularly from metallic restorations and enamel surfaces exposed to the oral mucosa. Dental hygienists administered professional oral care including patient education about self-care at least once a week. Professional oral care methods included mechanical tooth cleaning to remove dental plaque, and gentle removal of mucosal debris with a water-drenched sponge to keep the oral cavity as clean as possible. The professional oral care was continued until the end of the radiotherapy treatment or hospital discharge.

### 2.6. Statistical Analysis

Patient characteristics were assessed for differences in radiation-induced mucositis severity (grade 1 or 2 vs. grade 3 or more). The exploratory variables analyzed in the univariate logistic regression model were included in the multivariate logistic regression model and assessed as follows: age; BMI; neutrophil and lymphocyte count; NLR; concentrations of serum albumin, hemoglobin, and serum creatinine (continuous for univariate analysis to assess linear trends); sex (male vs. female); diabetes mellitus (with vs. without); ECOG PS (0, 1 vs. 2, 3); primary tumor location (nasopharynx vs. oropharynx); stage (I or II vs. III or IV); concomitant CRT (radiotherapy alone vs. BRT or CRT); and radiation method (3D-CRT vs. IMRT). Baseline variables with *p* < 0.20 in the univariate analysis and two variables (therapy [8] and radiotherapy method [2]) known to have an influence on the severity of mucositis were included in the multivariable models.

All statistical analyses were performed using Statistical Package for the Social Sciences for Windows (version 25; IBM Corp., Tokyo, Japan). Statistical significance was defined as a two-sided *p*-value < 0.05.

## 3. Results

### 3.1. Patients

One hundred patients were included in this retrospective study. Grade 3 radiation-induced mucositis occurred in 47 patients (47%). None of the patients developed grade 4 or 5 mucositis. Table 1 shows the patient details, tumor characteristics, therapy approaches, and laboratory test results immediately before radiotherapy. Blood examination data were obtained before radiotherapy. The median time interval was 15 days (interquartile range: 0.5–33.5) in the case of radiotherapy. For chemoradiotherapy, the median time interval was 1 day (interquartile range: 0–7). Almost all of the radiation dose distribution was limited to the primary tumor site and the bilateral neck.

### 3.2. Association of Exploratory Variables with Grade 3 Mucositis

Table 2 provides the results of the univariate analysis. The following variable was found to be significantly associated with the occurrence of grade 3 mucositis: lymphocyte count (OR = 0.41; 95% CI = 0.20–0.86; *p* = 0.018). Table 3 presents the multivariate analysis results. Lymphocyte count was significantly associated with grade 3 mucositis (OR = 0.40; 95% CI = 0.19–0.86; *p* = 0.018).

## 4. Discussion

This retrospective study investigated the outcomes for grade 3 mucositis during radiotherapy for nasopharyngeal or oropharyngeal cancer with adjustment for clinical characteristics. The results showed that the incidence of grade 3 mucositis was 47% and the development of grade 3 mucositis was significantly associated with a lower lymphocyte count.

A prior study showed that low leukocyte or lymphocyte counts were found to be significantly associated with severe oral mucositis in 326 patients who underwent radiotherapy for oral and oropharyngeal carcinomas [9]. Included in these were patients who underwent postoperative radiotherapy or postoperative CRT. In the present study, we excluded patients who underwent postoperative radiotherapy or postoperative CRT because we thought that the postoperative condition affected the severity of mucositis via vascular insufficiency. As a result, even though patients who underwent postoperative radiotherapy or postoperative CRT were excluded, we found that a lower lymphocyte count was significantly associated with grade 3 mucositis.

Our previous study showed that the NLR was significantly associated with grade 3 radiation-induced mucositis in hypopharyngeal or laryngeal cancer patients [6]; however, the NLR was not associated with grade 3 mucositis in nasopharyngeal or oropharyngeal cancer patients. It is likely that the difference is due to the type of carcinoma. Almost all head and neck cancers are squamous cell carcinomas, and the most important risk factors are tobacco and alcohol consumption [1]. Moreover, two important human oncoviruses, Epstein–Barr virus (EBV) and human papillomavirus (HPV), are causally associated with nasopharyngeal and oropharyngeal cancers, respectively [10]. Both cancers are responsive to radiation [11]. HPV-positivity has been recognized as a single independent favorable prognostic factor for head and neck cancer survival [12], even for patients with recurring or metastatic cancer [13]. In this study, the association between p16 and grade 3 radiation-induced mucositis was not analyzed because one-third of the oropharyngeal cancer patients’ p16 type was unknown (Table 1). In addition to the difference in the types of carcinoma, there are differences in the radiation fields or the organ function which are thought to affect the severity of mucositis. Further research is required to clarify the association between the type of carcinoma and the severity of mucositis.

Another study showed that there was a close relationship between high NLR values and the risk of developing severe oral mucositis in patients irradiated due to head and neck cancer [14]. The study included 161 patients (78%) who underwent postoperative radiotherapy or CRT. Preoperative neutrophil and lymphocyte counts, and NLR values, were different from the postoperative values in patients with head and neck cancer [15]; additionally, the preoperative lymphocyte counts dynamically changed after surgery in patients with esophageal squamous cell carcinoma [16]. These data suggest that pre-radiation blood test values were important to predict the severity of radiation-induced mucositis.

Many studies have reported that tumors may be infiltrated by lymphocytes. These findings have suggested that the lymphocytes identify and destroy malignant cells [17,18]. Lower pre-treatment lymphocyte count predicts worse survival in breast cancer, colorectal cancer, and lung cancer [19,20,21]. Lymphopenia prior to initiating treatment has been associated with poor prognosis in patients with esophageal squamous cell carcinoma [22]. Lymphopenia prior to radiotherapy was suggested to predict severe mucositis as the NLR has previously been reported to be a strong predictor of mortality in head and neck cancer patients [23,24], and to be significantly associated with severe radiation-induced mucositis in pharyngeal or laryngeal cancer patients [6].

High NLR can result from lower lymphocyte counts. Lymphocytes require host immunocompetence and lymphocyte counts may reflect nutritional status. In fact, the controlling Nutritional Status (CONUT) score, a screening tool for evaluating malnutrition, takes into account serum albumin, total cholesterol, and total lymphocyte levels [25]. As such, the relationship between lymphocyte count and nutrition may explain the therapeutic benefits of oral glutamine supplementation in preventing and ameliorating radiation-induced oral mucositis among patients with head and neck cancer, as described previously [26].

This study has several limitations: it is a single-center retrospective study with a small number of patients; therefore, it is unclear whether the results obtained can be generalized. However, the main strength of our study is that the study population was most likely homogeneous because all patients underwent definitive radiotherapy or CRT for nasopharyngeal or oropharyngeal cancer and did not receive surgery before radiotherapy.

High NLR is a well-known predictor of poor cancer prognosis. Our previous study showed that high NLR indicated severe radiation-induced mucositis in pharyngeal or laryngeal cancer patients. In this study, lower lymphocyte counts are suggested to predict severe radiation-induced mucositis in nasopharyngeal or oropharyngeal cancer patients. Additionally, severe radiotherapy-induced mucositis does not clearly predict a good prognosis in patients with nasopharyngeal or oropharyngeal cancer. Thus, the association between severe mucositis and overall survival, disease-free survival, or progression-free survival should be identified.

## 5. Conclusions

The findings of this study suggest that lower lymphocyte counts pre-radiation are a predictive risk factor for severe mucositis in patients who undergo definitive radiotherapy or CRT for nasopharyngeal or oropharyngeal cancer.

## Figures and Tables

**Table 1:** Patient characteristics.

		Radiotherapy-Induced Mucositis				
		Grade 1 and 2			Grade 3	
		N = 53			N = 47	
Characteristic	Category	N	%		N	%
Primary tumor location	Nasopharynx	10	19		12	26
	Oropharynx	43	81		35	74
Histopathology	Nasopharynx					
	Squamous cell carcinoma	10	100		8	67
	Differentiation of squamous cell carcinoma			N		
	Keratinizing			1		
	Non-keratinizing			11		
	Basaloid			3		
	Unknown			3		
	Lymphoepithelioma	0	0		4	33
	Oropharynx					
	Squamous cell carcinoma	43	100		35	100
	Differentiation of squamous cell carcinoma			N		
	Keratinizing			18		
	Non-keratinizing			21		
	Basaloid			0		
	Unknown			39		
	p 16 protein			N		
	Positive			27		
	Negative			21		
	Unknown			30		
Sex	Male	45	85		36	77
	Female	8	15		11	23
Age	Median (25–75% tile)	66.0 (62.0–74.0)			63.0 (57.5–71.0)	
BMI (kg/m^2^)	Median (25–75% tile)	21.3 (19.4–24.9)			21.8 (19.4–24.8)	
ECOG PS	0	25	47		28	60
	1	20	38		12	25
	2	8	15		7	15
Diabetes mellitus	Without	43	81		42	89
	With	10	19		5	11
Stage	1	7	13		6	13
	2	8	15		2	4
	3	11	21		10	21
	4	27	51		29	62
Treatment	Radiotherapy alone	13	25		7	15
	Bioradiotherapy	5	9		9	19
	Chemoradiotherapy	35	66		31	66
Total radiation dose	Median (25–75% tile)	70.0 (70.0–70.0)			70.0 (68.0–70.0)	
Radiotherapy method	IMRT	39	74		37	79
	3D-CRT	14	26		10	21
Completion of radiotherapy	Completion	50	94		42	89
	Discontinuation	3	6		4	9
	Treatment pause	0	0		1	2
Neutrophil count (×10^3^/dL)	Median (25–75% tile)	4.0 (2.7–5.0)			4.0 (2.9–5.5)	
Lymphocyte count (×10^3^/dL)	Median (25–76% tile)	1.7 (1.3–2.2)			1.4 (1.2–1.8)	
NLR	Median (25–75% tile)	2.2 (1.4–3.2)			2.6 (1.8–4.0)	
Serum albumin (g/dL)	Median (25–75% tile)	3.7 (3.4–4.0)			3.7 (3.5–4.1)	
Hemoglobin (mg/dL)	Median (25–75% tile)	13.2 (11.9–14.2)			12.9 (11.8–13.9)	
Serum creatinine (mg/dL)	Median (25–75% tile)	0.85 (0.73–0.96)			0.83 (0.69–0.92)	

Abbreviations: BMI, body mass index; ECOG PS, Eastern Cooperative Oncology Group performance status; BRT, bioradiotherapy (cetuximab with radiotherapy); CRT, chemotherapy (cisplatin or carboplatin with radiotherapy); IMRT, Intensity-modulated radiotherapy; and 3D-CRT, Three-dimensional conformal radiotherapy NLR, neutrophil-to-lymphocyte ratio.

**Table 2:** Univariate analysis of association between grade 3 mucositis and clinical characteristics in patients underwent radiotherapy for nasopharyngeal or oropharyngeal cancer.

Variable	Category (Reference)	OR	95% CI	*p*-Value
Sex	Male (Female)	0.58	0.21–1.60	0.294
Age		0.97	0.94–1.01	0.126
BMI (kg/m^2^)		1.00	0.89–1.11	0.930
ECOG PS	≥2 (0 or 1)	0.98	0.33–2.96	0.978
Diabetes mellitus	With (Without)	0.51	0.16–1.62	0.256
Primary tumor location	Nasopharynx (Oropharynx)	1.47	0.57–3.81	0.423
Stage	III, IV (I, II)	1.92	0.73–5.06	0.185
Therapy	BRT or CRT (Radiotherapy alone)	1.86	0.67–5.14	0.233
Radiotherapy method	IMRT (3D-CRT)	1.33	0.53–3.36	0.549
Total radiation dose (Gy)		1.00	0.89–1.12	0.960
Neutrophil count (×10^3^/dL)		0.92	0.77–1.10	0.370
Lymphocyte count (×10^3^/dL)		0.41	0.20–0.86	0.018
NLR		1.00	0.85–1.17	0.960
Serum albumin		0.88	0.37–2.08	0.774
Hemoglobin		0.98	0.79–1.21	0.835
Serum creatinine		0.28	0.04–2.08	0.215

Abbreviations: BMI, body mass index; ECOG PS, Eastern Cooperative Oncology Group performance status; NLR, neutrophil-to-lymphocyte ratio; BRT, bioradiotherapy (cetuximab with radiotherapy); CRT, chemotherapy (cisplatin or carboplatin with radiotherapy); IMRT, Intensity-modulated radiotherapy; 3D-CRT, Three-dimensional conformal radiotherapy; OR, odds ratio; and CI, confident interval.

**Table 3:** Multivariate logistic regression analysis of risk factors for grade 3 mucositis in patients underwent radiotherapy for nasopharyngeal or oropharyngeal cancer.

Risk Factor	Category (Reference)	OR	95.0% CI	*p*-Value
Lymphocyte count (×10^3^/dL)		0.40	0.19–0.86	0.018
Age		0.98	0.94–1.02	0.301
Stage	III or IV (I or II)	1.56	0.53–4.56	0.416
Therapy	BRT or CRT (Radiotherapy alone)	1.16	0.33–4.11	0.820
Radiotherapy method	IMRT (3D-CRT)	1.51	0.54–4.21	0.435

Abbreviations: BRT, bioradiotherapy (cetuximab with radiotherapy); CRT, chemotherapy (cisplatin or carboplatin with radiotherapy); IMRT, Intensity-modulated radiotherapy; 3D-CRT, Three-dimensional conformal radiotherapy; OR, odds ratio; and CI, confident interval.

## Data Availability

The data presented in this study are available on request from the corresponding author.
